# Early ERP Evidence for Children’s and Adult’s Sensitivity to Scalar Implicatures Triggered by Existential Quantifiers (*Some*)

**DOI:** 10.3389/fpsyg.2021.657408

**Published:** 2021-09-10

**Authors:** Daniele Panizza, Edgar Onea, Nivedita Mani

**Affiliations:** ^1^Department of English Studies, University of Göttingen, Göttingen, Germany; ^2^Courant Research Centre “Text Structures”, University of Göttingen, Göttingen, Germany; ^3^Department of German Studies, University of Graz, Graz, Austria; ^4^Psychology of Language Research Group, University of Göttingen, Göttingen, Germany; ^5^Leibniz ScienceCampus Primate Cognition, Göttingen, Germany

**Keywords:** pragmatics, implicatures in language acquisition, implicature, developmental pragmatics, pragmatic inferencing, speech processing, N400, scalar implicature

## Abstract

How quickly do children and adults interpret scalar lexical items in speech processing? The current study examined interpretation of the scalar terms *some* vs. *all* in contexts where either the stronger (*some* = *not all*) or the weaker interpretation was permissible (*some* allows *all*). Children and adults showed increased negative deflections in brain activity following the word *some* in *some*-infelicitous versus *some*-felicitous contexts. This effect was found as early as 100 ms across central electrode sites (in children), and 300–500 ms across left frontal, fronto-central, and centro-parietal electrode sites (in children and adults). These results strongly suggest that young children (aged between 3 and 4 years) as well as adults quickly have access to the contextually appropriate interpretation of scalar terms.

## Introduction

While communicating, interlocutors often derive additional interpretations from utterances that are not directly encoded in the semantics of the words they use. Such interpretations are obtained by performing additional semantic/pragmatic operations to the lexical meaning of these words and the way they cohere at the sentence level. Pragmatic inferencing, as this phenomenon is called, is pervasive in language use. The most studied case of pragmatic inferencing, potentially due to the systematicity and robustness of this class of pragmatic inferences, is that of scalar implicatures.

The standard view of scalar implicatures, inspired by Grice’s seminal work (Horn, 1973; Grice, 1975; Levinson, 2000), holds that such inferences are triggered by the use of a lexical item that is a member of an asymmetrical entailing scale (i.e., a Horn scale). For instance, let us consider the exchange presented in (1) below:

(1) (a) Speaker A: What did you eat?
  (b) Speaker B: I ate some of the apples on the table.

Speaker B’s answer involves the word *some*, which is a member of the Horn scale <*some*, *all*>, with *all* being more informative–i.e., logically stronger–than *some.* This relationship, which holds for any Horn scale, can be illustrated as follows. Consider the sentence in (2), which includes the same words as (1b) with the exception of the quantifier *some* that has been substituted by *all* in (2).

(2) I ate all of the apples on the table.

Sentence (2) is logically stronger than (1b) because the latter unilaterally entails the former. This means that in every situation in which (2) is true, (1b) must be true as well but not the other way around. This is shown by the validity of the following material implication: if I ate all the apples on the table I also ate some of them. Instead, the converse does not hold, namely if I ate some of the apples on the table I did not necessarily eat all of them. From this considerations we may conclude that *all* is logically stronger than *some.*

The scalar algorithm proceeds as follows: Since Speaker A assumes that Speaker B obeys the Gricean Maxims and Principle of Communication, she knows that Speaker B made her contribution as informative as required for the purpose of the conversation. Hence, from the fact that Speaker B uttered *some*, she infers that Speaker B was not in the position of uttering the same sentence including a stronger item from the scale (*all*) as in (2).

From this reasoning, Speaker A concludes–i.e., conversationally implicates–that the proposition in (2) is false. This last step represents the informational content of the implicature. Once the negation of (2) is added to the original assertion (1b) it leads to an interpretation that is more informative than the one without the implicature. The result of this process is the meaning in (3):

(3) I ate some of the apples on the table and I did not eat all of them.

We may rephrase this process in the following terms, which represents standard terminology in the field: the semantics of the weak scalar item, *some*, originally compatible with the meaning of the stronger item (i.e., Lower-bounded meaning of *some* = “some and maybe all”) receives an upper-bound (i.e., Upper-bounded meaning of *some* = “some but not all”) through the scalar implicature, as in (3).

Scalar inferencing is a pervasive phenomenon that does not only affect the interpretation of quantifiers such as *some*, but also that of adjectives, verbs, phrasal connectives, and other linguistic dimensions (cf. Horn, 1973; Levinson, 2000). Consider the following examples:

(4) It’s cold outside, but it’s not freezing.
(5) The children hit the window glass with a ball but they didn’t break it.
(6) Mary will buy a new car or a motorbike but not both.

The examples in (4–6) involve Horn scales composed by adjectives (<*cold*, *freezing*>), verbs (<*hit*, *break*>) and connectives (<*or*, *and*>). All these scales share the property mentioned above, namely, every element of the scale is logically stronger than the elements to its left. This can be shown, once again, by the validity of the following conditional statements: if it’s freezing it is also cold, if the children broke the glass with a ball they also hit it, if Mary bought both a car and a motorbike she also bought one of the two. The effect of a scalar implicature computed through these scales is exactly the same as in the case of the inference triggered by *some* in (3): the assertion of a weak element of the scale conversationally implicates that the same sentence including a stronger element does not hold.

While theories differ on how exactly this process takes place in the minds of speakers (cf. Sperber and Wilson, 1985/1995; Levinson, 2000, for an overview), any account of scalar implicatures maintains that something very similar to the algorithm described above occurs in order for scalar implicatures to behave the way they do. That is, they are triggered by certain lexical items through a semantic/pragmatic mechanism, they enrich the original sentence meaning leading to a more informative interpretation and, unlike other inferences such as semantic entailment or pragmatic presuppositions, they can be cancelled or suspended without any sense of contradiction (Levinson, 2000). For instance, in the dialogue in (1) Speaker B could have overridden the implicature by adding a continuation such as “…in fact I ate all of them!’’. Notice that the same principle applies to the other examples above (‘‘it is cold, in fact it’s freezing’’, ‘‘the children hit the window glass with the ball and they broke it’’; ‘‘Mary will buy a car or a motorbike, in fact she will buy both’’). These examples show that scalar implicatures are optional and they can be cancelled without contradicting the meaning of the original sentence.^1^

In the last decades, a number of experimental studies have investigated the comprehension and online processing of scalar implicatures in adults and children. The results from these studies have not always converged and have generated much debate as to how scalar implicatures are processed in our cognitive system, as well as a flourishing of processing models to account for the varied results. This debate regards the timing of scalar implicature generation (Huang and Snedeker, 2009a; Grodner et al., 2010), their cognitive cost (Bott and Noveck, 2004; Bott et al., 2012), whether they are encoded in some kind of lexical ambiguity or underspecification, and their relationship with other pragmatic inferences (Bergen and Grodner, 2012; Tieu et al., 2014; Bill et al., 2016).

Important for the purposes of the current study, however, are two main patterns of findings in the literature to-date: (a) young learners have been found to perform differently from adults–i.e., they show a systematically lower rate of scalarly strengthened interpretations in overt decision tasks up to 12 years of life (Katsos and Bishop, 2011), (b) no previous study has ever reported any evidence of children younger than 4-years of age displaying systematic sensitivity to scalar implicatures (cf. Barner et al., 2009; Huang et al., 2013).

Young children’s reported failure to derive scalar implicatures is unexpected given that children, from an early age, appear to possess sophisticated pragmatic skills. For instance, they are able to (a) understand that other speakers may have different perspectives on a common situation (cf. Theory of Mind in development, Csibra and Gergely, 2009; Rhodes and Brandone, 2014), (b) restrict the meaning of unknown words given their knowledge of other familiar words (cf. Principle of Contrast Clark, 1990; Halberda, 2003) and (c) adopt pragmatic strengthening in other dimensions such as numerals (Panizza et al., 2013) and *ad hoc* scales (Stiller et al., 2015; Foppolo et al., 2020).

Against this background, the current study will examine whether 3-year-old children are sensitive to classic scalar inferences triggered by existential qualifiers (i.e., *some* → *not all*) such as the example in (1), using the event-related potential (ERP) methodology. In the next paragraph we will outline the main motivation underlying this choice. Furthermore, to provide the reader with the background relevant to our experimental inquiry we will briefly illustrate the relevant studies investigating children’s comprehension of scalar inferences as well as adult ERP based findings.

## Developmental Literature on Pragmatic Inferencing

The notion of a systematic difference in how adults and children interpret scalar terms has emerged since the very first psycholinguistic studies adopting evaluative judgments on previously made assertions. Weak scalar terms such as *or* (Paris, 1973; Braine and Rumain, 1981), *might* (Noveck, 2001) and *some* (Smith, 1980; Noveck, 2001) have been found to be interpreted significantly more often according to their logical interpretation–i.e., without the scalar implicature–than with the strengthened interpretation in English and French children ranging from 4 to 9 years of life. Many subsequent studies employing overt judgments, performing actions (cf. Covered Box Task in Huang et al., 2013), the Truth Value Judgment task (cf. Crain and Thornton, 1998) or some variant thereof replicate this pattern. These studies generally involve a critical condition in which the participant is presented with a real situation or a visual scenario and must judge whether an underinformative statement describing this situation is right or not. For instance, the utterance “Kermit ate some of the cakes on the table” provides an underinformative description of a situation in which Kermit actually ate all of the cakes. Higher acceptance rates of underinformative statements were reported in studies investigating the logical connective *or* in 3- to 6-year-old children (Chierchia et al., 2001), verb predicates like *start* in 5-year-old children (Papafragou and Musolino, 2003), the indefinite *a* in 2-year-old children (Barner et al., 2009) and existential quantifiers like *some* in 2- to 7-year-old children (Papafragou and Musolino, 2003; Guasti et al., 2005; Pouscoulous et al., 2007; Katsos and Bishop, 2011; Huang et al., 2013).

However, in more recent studies, children have been found to successfully generate scalar implicatures if provided with some contextual or pragmatic facilitation, albeit not at the same rate as adults. Foppolo et al. (2012) report that 4- to 6-year-olds correctly reject 77.5% of underinformative statements with *some* in a reward task including highly salient expectations, cognitive gains and very accessible alternatives (cf. Guasti et al., 2005, for similar results with 7-year-old children). Pouscoulous et al. (2007) report that 64% of their 4-year-old participants were consistently producing implicatures with *quelques* (a French word for *some*) in an action-based task. Papafragou and Musolino (2003) boosted the rate of rejections of *some* and *start* from about 10% to about 50% in 5-year-olds by improving the experimental context and making the experimental goals easier. Foppolo et al. (2012) report an improvement from 40 to 72% of rejections of *some* in 5-year-olders when they were presented with two trials displaying a true and false scenario for the strong item (i.e., *all*) before the critical trial involving *some*. Similarly, Katsos and Bishop (2011) obtained a significant improvement of scalar implicatures with *some* in 5- to 6-year-old children, from about 30 to 70%, by switching the task from a binary (involving small/big strawberry) to a ternary Reward Task (small/big/huge strawberry).

Developmental studies on children’s interpretation of numerals add to this already complicated picture. There are few doubts that children consistently assign the upper-bounded interpretation to numeral quantifiers (i.e., *two* = “exactly two”). For instance, Barner et al. (2009) found that, already at the age of two, young learners assign the upper-bounded meaning to the numeral *one* but do not restrict the interpretation of the indefinite *a* computing the upper-bound. Moreover Huang et al. (2013) found that 3-year-old children are reluctant to assign the upper-bounded meaning to *some* but they do so with *two* at an adult-like level. They take this as support for the suggestion that numerals unlike scalar quantifiers lexically encode their upper-bound, as further suggested by other studies with adults (Panizza et al., 2009).

Different explanations have been proposed to account for increased acceptance of underinformative sentences including scalar items in children. On the one hand, some accounts maintain that this behaviour is due to the lack of computation of the implicature: Children’s failure at computing scalar implicatures can, in turn, be attributed to insufficient computational resources (Reinhart, 2004; Guasti et al., 2005), difficulty at retrieving and activating (Skordos and Papafragou, 2012) or lack of lexical knowledge (Chierchia et al., 2001; Barner et al., 2011) of the relevant alternatives. Thus, for instance, the differential ease with which children derive the upper-bound of numerals vs. scalar quantifiers is typically attributed to the fact that numeral alternatives are much easier to retrieve given the availability and high saliency of the number scale, whereas other scalar alternatives must be lexically acquired and contextually activated.

On the other hand, according to an alternative explanation, children’s acceptance of pragmatic violations remains compatible with the generation of a scalar implicature. According to this explanation, children nevertheless continue to accept underinformative statements due to their insufficient cognitive skills with respect to task-related demands (Papafragou and Musolino, 2003; Papafragou and Tantalou, 2004), higher tolerance to pragmatic infelicity (Katsos and Bishop, 2011), poor ability in changing strategy or to shift one’s perspective (Foppolo et al., 2012) and difficulty in conflict monitoring (Shetreet et al., 2014a). The findings reported above showing increased rejection of underinformative statements in young children given additional contextual information appear to be more in keeping with the latter explanation.

## Previous Neuroscientific Studies Investigating Scalar Implicatures

A few recent studies have investigated the processing of scalar implicatures during silent reading using the ERP methodology. Noveck and Posada (2003) reported a reduction in the N400 triggered by a critical word in underinformative statements (e.g., trunks in “Some elephants have trunks”) as compared to patently true and false statements (see also Nieuwland et al., 2010; Hartshorne et al., 2015).

The N400 effect is the most well-known neuropsychological correlate of semantic processing (Kutas and Hillyard, 1980). It is a negative deflection peaking at around 400 ms from the onset of the critical stimulus, triggered by a wide range of linguistic manipulations such as semantic anomalies, difficult contextual integration, unpredicted words and, in general, increased semantic processing demands (cf. Kutas and Federmeier, 2000, for a review). It is still under debate whether the nature of this effect is linguistic—triggered by difficulties in lexical access (Lau et al., 2008), violation of expectations on upcoming words (Kutas and Hillyard, 1984), unification of meanings (Hagoort, 2019)—or extra-linguistic, possibly due to difficulties in conceptual integration or an extensive search in long-term memory as a reaction to problematic interpretation (Kutas and Federmeier, 2000).

We focus, however, on ERP studies that have examined the processing of scalar quantifiers such as *some* in a picture-sentence verification task, involving a set-up similar to the one employed in our study. Politzer-Ahles et al. (2013) presented participants with a visual scenario, e.g., five girls sitting on a blanket suntanning, as they heard or read a sentence such as “in the picture, some of the girls are sitting on a blanket suntanning.” They manipulated the visual scenario (*only some* vs. *all* the girls were sitting on a blanket) and the quantifier (*some* vs. *all*) in order to create pragmatic (visual scenario-*all*, auditory-*some*) vs. semantic violations (visual scenario-*some*, auditory-*all*). Across two experiments they report a sustained negativity for pragmatic violations vs. a sustained positivity for semantic violations, with the effect of the pragmatic violation arising as early as 200 ms with a scalp distribution similar to the N400 effect. Panizza and Onea (2014) and Hunt et al. (2013) report a larger N400-like wave for semantic violations vs. a reduced N400 for pragmatic violations elicited by *some* in German and English, respectively. In the study by Panizza and Onea (2014) this effect were followed by a late left anterior negativity (L-LAN) elicited by pragmatic violations relative to a late frontal positivity (FP600) elicited by semantic violations. Spychalska et al. (2016) found a biphasic pattern composed by an N400 followed by a centro-parietal positivity for both semantic violations, and similar effects for pragmatic violations but only in the participants who consistently rejected the underinformative statements. L-LAN effects have been reported in studies investigating semantic violations such as logical contradictions (Shao and Neville, 1998; Steinhauer et al., 2010) and semantic revision (Baggio et al., 2008). FP600 effects have been reported in association with discourse complexity (Kaan and Swaab, 2003), semantic violations and repair (Friederici et al., 2002; cf. Panizza, 2012 for an overview).

Taken together, some of the ERP findings on scalar implicature violations highlight common neural signatures (i.e., N400-like effects) of linguistic anomalies due to pragmatic infelicity vs. semantic falsity while other results suggest different cognitive operations dealing with these anomalies, resulting in positivities associated with semantic falsity vs. negativities associated with pragmatic infelicity. Remarkably, the results showing opposite neurophysiological effects for semantic vs. pragmatic violations were found in experiments in which the ERPs were time-locked to the quantifier (Politzer-Ahles et al., 2013; Panizza and Onea, 2014).

One recent study presents some data that are potentially in conflict with the picture outlined above. Augurzky et al. (2019) employed a different experimental design which nonetheless bears some similarities with the present study. They presented two visual contexts simultaneously followed by a statement such as “Some dots are blue, which are on the right box” and recorded the ERPs time-locked with different words (i.e., *some*, *blue*, and *box*). The authors report a reduced N400 effect in the ERPs recorded at the colour adjective (e.g., *blue*) for the condition involving both boxes displaying a pragmatic violations (e.g., all dots were blue in both boxes). Instead, they found a larger N400 for the mixed condition, with one box displaying a pragmatic felicitous scenario (some but not all of the dots were blue) and the other displaying a pragmatic violation. The largest N400 was detected in the false condition, where all the boxes were of the opposite colour. These results are at odds with the pattern emerging from the two studies discussed above in that both conditions including a pragmatic violation showed a smaller N400 effect compared to the condition featuring a semantic violation. Yet, the difference in the paradigm as well as the absence of a control condition where only a subset of the dots (“some but not all”) appear in one colour in both boxes, i.e., both boxes display a felicitous scenario, make it difficult to compare their results with the present study.

Finally, Shetreet et al. (2014a, b) conducted an fMRI experiment adopting the sentence-picture verification task with adults and children. With adults, they found two separate brain networks selectively sensitive to implicature generation vs. mismatch. A subregion of the Inferior Frontal Girus (IFG, BA 47) was more active when the implicature was supported by the visual context whereas the left Middle Frontal Girus (MFG) and Medial Frontal Girus (MeFG), which is associated to high-order cognitive functions, was more active only in the mismatch condition (i.e., implicature violation). In 5- to 6-year-old children, they found an effect of an increased activation of the IFG for implicature computation but, critically, no difference in the MFG and a deactivation of the MeFG in correspondence with implicature mismatch. This suggests that children do derive scalar implicatures with *some*, and their tolerance to pragmatic violations is due to differences in the processing, or lack thereof, of the implicature mismatch as compared to older children and adults.

## The Present Study

The studies to-date suggest that young children often fail at restricting the meaning of scalar quantifiers through pragmatic strengthening, but succeed at doing so with numerals (Huang et al., 2013; Panizza et al., 2013), *ad hoc* implicatures (Stiller et al., 2015; Foppolo et al., 2020) whilst also mastering their understanding and use of the principle of contrast, another kind of pragmatic inferencing, to acquire new words. Furthermore, given that 3-year-old children are competent in other tasks requiring sophisticated pragmatic skills like theory of Mind (Onishi and Baillargeon, 2005), counterfactual thinking (Harris et al., 1996) it would only be reasonable to expect that they ought to be able to generate scalar implicatures with similar ease. This expectation holds, unless we admit that there is something specific to scalar implicatures derived from quantifiers that makes processing of such implicatures more difficult, for example–as proposed by Barner et al. (2011)–the lack of lexical knowledge that links the stronger alternative (i.e., *all*) to the scalar trigger *some*. Against this background, the current study will evaluate the claim that children possess full competence for deriving scalar inferences at a very young age using the ERP methodology. In particular, we will examine the processing of scalar implicatures at a younger age than has been reported in the literature to-date (the youngest participants showing competence at deriving scalar implicatures to-date were 4-year-old in Pouscoulous et al., 2007 and Papafragou and Tantalou, 2004), namely at 3-years of age. Given the difficulty of testing children’s performance with scalar inferences with overt decision tasks such as the Truth Value Judgment task, and given that even older children often fail at judging the pragmatic infelicity of sentences involving scalar terms, we believe that the ERP methodology is the optimal choice to tackle this issue. Since the ERP method places almost no demands on children’s behaviour and delivers a reliable measure of the brain mechanism underlying the infants’ language processing skills (cf. Männel and Friederici, 2008), it ought to provide us with more reliable information regarding children’s generation of scalar implicatures, in particular addressing the question whether 3-year-old children derive implicatures but fail at judging pragmatic violations or whether they fail at computing them altogether.

We use a single referent-auditory stimulus matching task, a simple version of the picture-sentence matching task used in adult studies investigating scalar implicatures (cf. Politzer-Ahles et al., 2013; Panizza and Onea, 2014; Shetreet et al., 2014a; Degen and Tanenhaus, 2015), combined with the ERP paradigm. Here, participants are presented with an image of two animals, e.g., a frog and a hedgehog side-by-side on the screen. Prior to the onset of the auditory stimulus, some (three of four) or all (four of four) objects immediately beneath one of the animals, e.g., the frog, move from beneath the frog to the hedgehog. Participants’ attention is then directed to the hedgehog with the question *Has the hedgehog all the keys?* [*Hat der Igel alle Schlüssel?*], followed by the answer *He has some of them* [*Er hat ein paar davon*].

There are a number of advantages to the simplicity of the above design which we highlight next: First, as noted above, the use of ERPs allows us to directly measure the brain’s immediate responses to the appropriateness of a linguistic expression to a single visually presented scene as opposed to tapping into children’s and adults’ metalinguistic judgement of the felicity of the pairing of an image and a spoken utterance (Chierchia et al., 2001; Barner et al., 2011; Skordos and Papafragou, 2012).

Second, the use of question-answer pairs makes the Question Under Discussion (“has the hedgehog all the keys?”) explicit: By making the scalar quantifier the focus of the answer (cf. Roberts, 2012), we overtly present the strong alternative before the scalar trigger (i.e., *all*), and create a strong informational contrast between the strong quantifier in the question and the weak quantifier in the answer. In simple terms, it constitutes a very efficient way to enhance the scalar inference originated by *some* (cf. Yang et al., 2018, for a study that uses the same strategy to enhance the derivation of scalar implicatures).

Third, the use of the question-answer pairs allows us an opportunity to contrast children’s responses to violations introduced by the critical word *some* (“*ein paar*”) versus mismatches introduced by the word *all*, (“*alle*”) in the question preceding the test sentence. That is, hearing the word *all* upon being presented with *some*-felicitous images, i.e., images where the hedgehog had three of four keys (the frog has the remaining key), should trigger a violation of a lexical expectation relative to *some*-infelicitous images. This is because when listeners are presented with a *some*-infelicitous visual context, where the hedgehog has all of the keys (i.e., an *all*-situation), they are predicted to pre-activate the lexical item *all* while hearing “has the hedgehog all the keys?”. Instead, when listeners are presented with a *some*-felicitous context they are not expected to pre-activate the quantifier *all* in that the visual scenario does not represent an *all-*situation.

In contrast, hearing the word *some* following exactly these images should trigger a violation in the opposite direction, i.e., hearing the word *some* upon being presented with a *some*-infelicitous image, where the hedgehog had all the keys (the frog has no keys) should trigger a pragmatic violation relative to *some*-felicitous images. Let us remark that the ERPs recorded at the presentation of *some* constitute the focus of the current experiments and are in all respects comparable to the pragmatic violation condition of previous studies such as Politzer-Ahles et al. (2013). The ERPs recorded at the onset of *all*, instead, only represents a control for lexical expectations, in that, here, the critical word occurs embedded in a question that always precedes the statement including *some*. For this reason the ERPs elicited by *all* substantially differ from those included in semantic violation conditions of previous studies. In what follows, we outline our predictions for the ERP components in more detail.

### Predictions on ERP Components

The earliest component that could be affected by our manipulation is the Phonological Mismatch Negativity (PMN), which is thought to reflect phonological processing sensitive to the expectations raised by the prior semantic context (Connolly and Phillips, 1994; D’Arcy et al., 2000; Newman et al., 2003). This component typically occurs with a frontocentral distribution (Connolly et al., 1990, 1992) although studies have reported a more widespread distribution of the PMN (Connolly and Phillips, 1994) and other works (cf. Lewendon et al., 2020) cast doubts on the existence of a reliable difference between PMN and N400 effects.

Another component relevant to our study is the N400, which typically occurs with a centroparietal distribution in the visual modality (Kutas and Van Petten, 1988) and a frontocentral distribution in the auditory modality (Connolly et al., 1990, 1992). Both these components have also been reported in previous studies with young children (Friedrich and Friederici, 2005; Sheehan et al., 2007; Mani et al., 2012). Sheehan et al. (2007) report finding an early (200–400 ms) and later negative component (400–600 ms) influenced by the congruence of an auditorily presented word as a label for a visually presented image, with a more negative going wave for incongruous word-image pairings, e.g., cup-book) compared to congruous pairings (e.g., cup–cup). The early time window (200–300 or 200–400 ms) also showed significant differences in the ERPs to correct and incorrect pronunciations of the labels of visually presented images in young children (Mills et al., 2004; Mani et al., 2012). Similarly, Friedrich and Friederici (2005) find early effects (between 150 and 400 ms) of semantic congruence of picture-word pairings (e.g., apple–apple vs. apple–book) in infants, with more negative responses to congruous words than incongruous (see also Mills et al., 2005; von Koss Torkildsen et al., 2006). Modulation of this component by semantic congruence at the sentence level is also reported in Hahne et al. (2004) and Holcomb et al. (1992) who examined 5- to 15-year-old children and reported similar N400 effects for all age-groups (see also Silva-Pereyra J. et al., 2005; Silva-Pereyra J. F. et al., 2005 abeit with a slightly delayed effect in 30-month-olds relative to 3- to 4-year-olds).

In the context of the current study, as noted above, if children and adults retrieve the stronger interpretation of *some*, i.e., “some but not all,” then ERPs to *some* in *some*-infelicitous videos should be distinct from ERPs to *some* in *some*-felicitous videos. Given that the participants hear exactly the same sentence in both conditions, any difference revealed by this contrast must be due to the effect of the visual context, i.e., animation they have seen prior to hearing the sentence. In this respect the two main questions we aim to address are the following: Are 3- to 4-year-old children sensitive to this manipulation, hence to pragmatic violations? If this is the case, is there any difference in latency and topography of the ERPs elicited by *some* in children vs. adults?

We are also interested in the ERPs time-locked to the onset of the word *all* in the question. Given that the *some-*infelicitous videos display a situation in which the depicted character deals with all the relevant objects in the scenario, this condition should raise the expectation of hearing a sentence including the quantifier *all*, e.g., by pre-activating its lexical representation. A very similar consideration can be drawn about expecting the quantifier *some* in the *some-*felicitous condition. Thus, if the effects revealed by this study are due to a violation of a lexical expectation—or the cognitive effort required to retrieve the quantifier when it has not been pre-activated (cf. Lau et al., 2008)—similar ERP profiles should be elicited by *some* [*ein paar*] in *some*-infelicitous vs. -felicitous conditions and by *all* [*alle*] in *some-*felicitous vs. -infelicitous conditions.

Alternatively, considering that that speakers tend to process and understand interrogative statements incrementally such as with declarative sentences (cf. Ueno and Kluender, 2009; Kao et al., 2010), hearing *all* in the question could generate a semantic mismatch in *some*-felicitous videos. Based on the results from previous studies (cf. Politzer-Ahles et al., 2013), thus, it is possible that ERPs measured at *some* vs. *all* in *some*-felicitous versus *some*-infelicitous videos should give rise to dramatically distinct neuropsychological profiles.

## Materials and Methods

### Participants

A total of 24 children (aged between 35 and 42 months, *M* = 37.29 m) and 24 adults (aged between 18 and 26 years, *M* = 22.33y; 17F and 7M) participated in the experiment. All participants came from families where German was the main language in use and lived in a German environment, although four children and three adults were bilingual and spoke a second language. The data from six children were not included in the final sample due to technical issues with data acquisition (1), noisy data (4) and the child not completing at least 50% of the experiment (1), leaving the data from 18 children for final analysis (8F and 10M). Children came from a sample of families who responded to an invitation letter sent to all families living with infants of appropriate age in the area. Adults were University students who received credits for participating in the experiment. The children were healthy, full-term infants without any pre- or perinatal complications. Adult and child participants had normal or corrected vision and no hearing problems. Parents gave informed consent for participation of their child in the study, while adult participants gave written consent before beginning the experiment and the researchers involved in the project ensured that the research was conducted in accordance with ethical guidelines of the Helsinki protocol.

### Materials

We created 120 audiovisual videos for use in the experiment. Each video lasted 10.5 s and followed the same format. The video began with the static presentation of two distinct familiar animals, e.g., a frog and a hedgehog, with between three to five objects underneath one of the animals, e.g., four keys under the frog. One second into the trial, some or all of the objects moved from this animal to the other, i.e., from under the frog to the hedgehog. The movement lasted until 4 s into the trial, i.e., a total of 3 s. The first auditory stimulus, i.e., the question *Has the hedgehog all the keys?* [*Hat der Igel alle Schlüssel?*] began such that the end of the question was 5,900 ms into the trial. Following a 1,500 ms pause, the second auditory stimulus, i.e., the first part of the answer *He had some of them* [*Er hat ein paar davon.*] began such that the beginning of the word *some* [*ein paar*] was 7,700 ms into the trial. To provide some indication of the continuity between the question and answer phase of the trial, the answer continued in either the affirmative or the negative–*He had some of them, therefore, yes* [*Er hat ein paar davon, also ja.*] or *He had some of them, therefore, no* [*Er hat ein paar davon, also nein.*]. To ensure that the affirmative or negative continuation did not systematically influence children’s responding in successive trials, half of *some*-felicitous images were followed by affirmative continuations (incorrect answer to the question) while the other half with negative continuations (correct answer to the question). Similarly, half of the *some*-infelicitous images were followed by negative continuations and the other half with affirmative continuations. Thus, importantly, children could not learn from the continuations whether *some* was felicitous to the image provided. The visual image remained on-screen for 625 ms after the offset of all auditory stimuli.

The 120 videos presented images of a total of 30 different animals (all reported to be known to individual children according to parental reports, an adaptation of the FRAKIS questionnaire). Thus each animal appeared in eight videos (two animals per video), paired with different animals: children never saw two animals paired together more than once. In addition, 20 different familiar objects (according to parental reports) were used across the 120 videos. Animals and objects appeared equally often in *some*-felicitous and *some*-infelicitous videos across videos. We also counter-balanced the position of the animal (targeted in the question and answer) on-screen, the number of objects on-screen, the direction of movement of the object (from the animal on the left to the animal on the right, or vice versa) across *some*-felicitous and *some*-infelicitous videos.

Auditory stimuli were spoken by a female native speaker of German in an engaging voice. To ensure that the physical stimuli to which the ERPs were triggered (*some* [*ein paar*]) were identical across conditions, we cross-spliced the answer from the beginning of the verb, i.e., from *has some of them*, *therefore yes/no* in both *some*-felicitous and infelicitous videos. Cross-splicing only began at the onset of the verb since animals are gender-marked in German and the response varied across *He has some…*, *She has some…*, and *It has some…* We further ensured the gender-marked article was identical across male, female and neutral sentences in *some*-felicitous and infelicitous videos. Similarly, the gender-marked article and animal label were cross-spliced into the question presented in the videos so that there was no physical difference in the scalar term (and other words common to all questions and answers) in both question and answer stimuli.

### Procedure

Each participant was presented with 120 trials in randomised order according to the format presented above. Of these, 60 trials were *some*-felicitous while 60 trials were *some*-infelicitous. *Some*-felicitous trials presented participants with the image of the frog and the hedgehog, where the frog retained some (one or two) of the keys and the hedgehog was in possession of the remainder of the keys (two, three, or four) at the end of the movement phase of the video. The utterance *He has some of them* in response to *Has the hedgehog all of the keys?* is therefore felicitous with the scalar interpretation of *some*.

Our critical trials were the *some*-infelicitous trials. Here, participants were presented with the image of the frog and the hedgehog, where the frog retained none of the keys at the end of the movement–all the keys were under the hedgehog at the end of the movement. The utterance *He has some of them* in response to *Has the hedgehog all of the keys?* is, therefore, infelicitous with the strong scalar interpretation of *some*, i.e., that *some* implies *not all*.

### ERP Data Acquisition and Analysis

After the electrode cap placement, children sat on their caregiver’s lap during the experiment 100 cm away from a projection screen (90 × 50 cm). Auditory stimuli were presented through two loudspeakers located immediately above the screen at an average of 65 dB. Visual stimuli (measuring 18.5 × 25 cm) were centrally located on the presentation screen.

Electrophysiological data was recorded using the Biosemi Active Two Amplifier system at a sampling rate of 2,048 Hz from 32 Ag/AgCl electrodes placed according to the 10–20 convention. Two additional electrodes were placed behind the ears (reference electrodes) and one additional electrode was placed under the left eye to be used for eye-artifact rejection. Electrode offsets were kept <25 μV. Electroencephalogram was re-referenced offline to the averaged mastoid reference. EEG data was filtered off-line using a 0.3 Hz high-pass forward filter and a 30 Hz low-pass (cf. Männel and Friederici, 2008, for guidelines on filtering data with children), zero-phase shift filter. Independent Component Analysis was performed with EEGLAB and components that were clearly identified as EOG artifacts were removed from the EEG (Delorme and Makeig, 2004). The remaining blink and movement artifacts were automatically rejected using an amplitude criterion adjusted to individual participants (constant at 150 μV for adult participants, between 150 and 300 μV for children). The topographic maps have been generated with the erpR package for R (Arcara and Petrova, 2014).

We defined two time-points in each trial that were of interest to us. The first centred around the critical word, alle, in the question, e.g., “Hat der Igel alle…”. The second centred around the scalar term, *ein paar*, in the response, e.g., “Er hat ein paar davon, also…”. Epochs were defined from -200 to 1,000 ms from the onset of the critical words in the question and the response, i.e., from the onset of the term *alle* (*all*) and *ein paar* (*some*). Baseline correction was performed in reference to pre-stimulus activity (-200 to 0 ms). Participants were excluded from the analysis if they provided fewer than 25 trials per condition following artefact rejection.

For analysis, we focussed on three non-overlapping time windows targeting the PMN (100–300 ms) and N400 (300–500 ms) components, and late negativities or positivities (500–700 ms). Repeated measures ANOVAs were performed separately for the data acquired at the midline electrodes and those acquired at the lateral ones to allow for quantitative analysis of hemispheric differences. The ANOVAs computed on the central line adopted condition (*some*-felicitous vs. *some*-infelicitous) and channel (one level for each one of the four midline electrodes: Fz, Pz, Cz, and Oz) as factors. Data acquired over the lateral electrodes were split across regions and averaged according to hemisphere yielding averaged values for left (F3 and F7) and right frontal (F4 and F8), left (FC1 and FC5) and right fronto-central (FC2 and FC6), left (C3 and T7) and right central (C4 and T8), left (CP1 and CP5) and right centro-parietal (CP2 and CP6) and left (P3 and P7) and right parietal (P4 and P8) electrodes. The 5 × 2 × 2 ANOVAs performed on these groups adopted region (frontal, fronto-central, central, centro-parietal, and parietal), hemisphere (left and right) and condition (same levels as above) as factors. The Greenhouse and Geisser (1959) correction was applied as required.

## Results

### Children (*All*)

**FIGURE 1:**
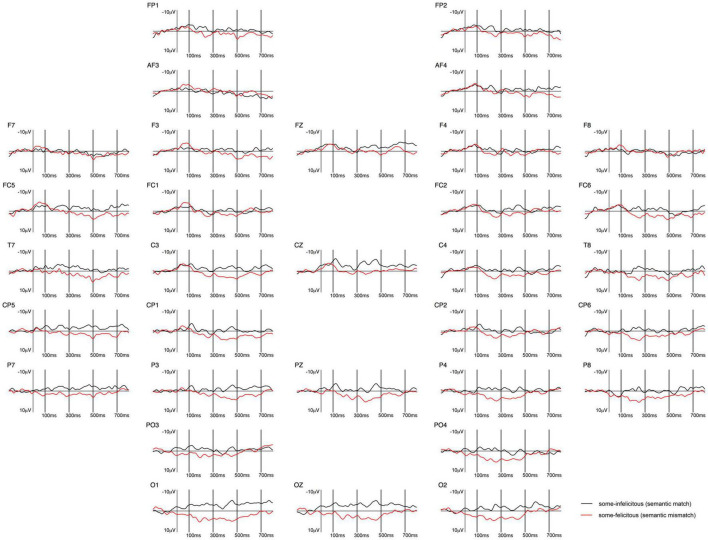
Grand-averaged ERPs recorded in children, time-locked to the presentation of the word “Alle” (*all*) for the *some*-infelicitous condition (black line) compared to the *some*-felicitous condition (red line).

**FIGURE 2:**
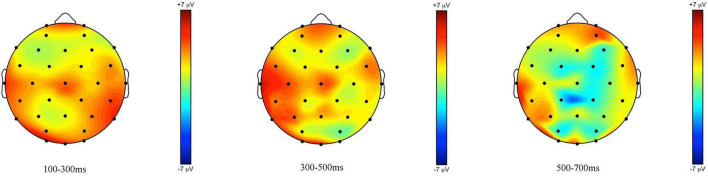
Topographic maps of the ERPs recorded in children, time-locked to the presentation of the word “Alle” (*all*) for the *some*-felicitous condition minus the *some*-infelicitous condition in the three time windows.

The ERPs measured at the onset of the quantifier *all* suggested an early but sustained positivity in the *some*-felicitous conditions compared to the *some*-infelicitous ones (cf. Figures 1, 2), more prominent across centro-parietal electrode sites, and a centrally distributed positivity between 300 and 500 ms in the *some*-felicitous condition relative to the *some*-infelicitous condition.

Repeated measures ANOVAs on the early time window (100–300 ms) yielded a significant interaction between condition × region on lateral electrode sites, *F*(4,14) = 2.39, *p* = 0.048, ηp2 = 0.15. Pivoting on region, further analyses found significant differences in brain activity to *some*-felicitous and infelicitous trials across central, *F*(1,17) = 7.85, *p* = 0.012, ηp2 = 0.32, and parietal electrode sites, *F*(1,17) = 6.33, *p* = 0.022, ηp2 = 0.27. Analyses on central-line electrode sites revealed a significant main effect of condition with brain activity being more positive to *some*-felicitous than infelicitous trials across central-line electrodes, *F*(1,17) = 4.67, *p* = 0.045, ηp2 = 0.22.

In the 300–500 ms time window, analyses of the lateral electrodes revealed no significant interactions or main effects of condition, ps > 0.1. However, analyses on central-line electrode sites revealed a significant main effect of condition with ERPs being more positive in *some*-felicitous than infelicitous trials across central-line electrodes, *F*(1,17) = 6.74, *p* = 0.019, ηp2 = 0.28. Analyses of the later time window revealed no significant main effects of condition or interactions with condition on either lateral or central-line electrodes.

### Children (*Some*)

**FIGURE 3:**
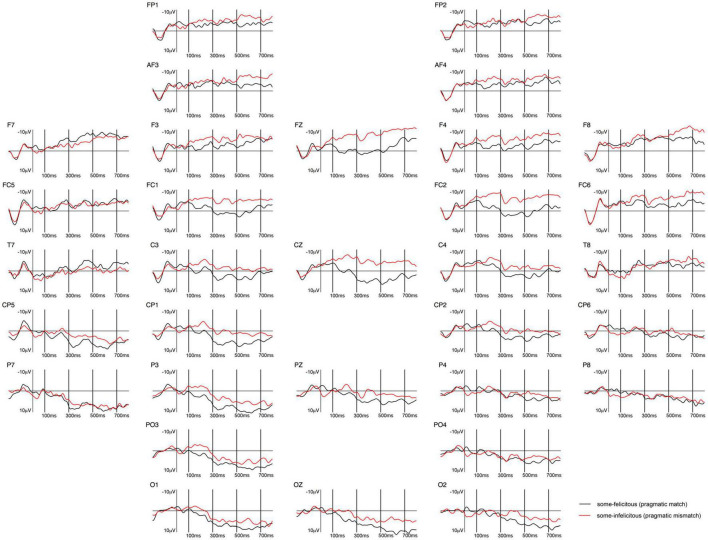
Grand-averaged ERPs recorded in children, time-locked to the presentation of the word “Ein paar” (*some*) for the *some*-felicitous condition (black line) compared to the *some*-infelicitous condition (red line).

**FIGURE 4:**
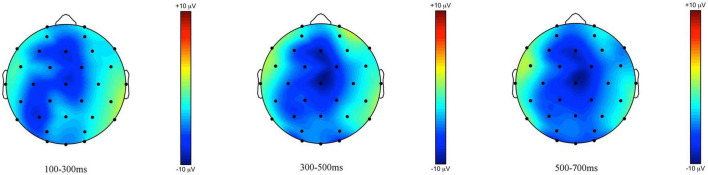
Topographic maps of the ERPs recorded in children, time-locked to the presentation of the word “Ein paar” (*some*) for the *some*-infelicitous condition minus the *some*-felicitous condition in the three time windows.

ERPs measured at the onset of *some* in children generated a sustained negativity with early onset in the *some*-infelicitous condition, relative to the *some*-felicitous condition, which reaches its peak on central line and centro-parietal electrode sites (cf. Figures 3, 4).

Repeated measures ANOVAs on the early time window yielded a near-significant interaction between condition × region × hemisphere on lateral electrode sites, *F*(4,14) = 2.95, *p* = 0.067, ηp2 = 0.15. Further analyses found no significant modulation of brain activity by condition in this early time window, ps > 0.1. However, analyses on central-line electrode sites revealed a significant main effect of condition with brain activity being more negative to *some*-infelicitous than felicitous trials across central-line electrodes, *F*(1,17) = 5.43, *p* = 0.034, ηp2 = 0.24.

In the 300–500 ms time window, analyses of the lateral electrodes did not reveal any significant effect. Pivoting on hemisphere, analyses of left hemisphere electrode sites found a significant interaction between condition × region, *F*(4,14) = 2.61, *p* = 0.043, ηp2 = 0.13, which we broke down further to reveal more negative ERPs to *some*-infelicitous trials relative to *some*-felicitous trials across left centro-parietal electrode sites, *t*(17) = 2.447; *p* = 0.026. No significant effects were found on analyses of right hemisphere electrode sites. However, analyses on central-line electrode sites revealed a significant main effect of condition with ERPs being more negative in *some*-infelicitous than felicitous trials across central-line electrodes, *F*(1,17) = 8.2, *p* = 0.011, ηp2 = 0.32.

In the 500–700 ms time window, analyses of the lateral electrodes yielded a significant interaction between condition × region × hemisphere on lateral electrode sites, *F*(4,14) = 3.13, *p* = 0.02, ηp2 = 0.16. Pivoting on region, we found a near-significant interaction between condition × region on fronto-central electrode sites, *F*(1,17) = 4.02, *p* = 0.061, ηp2 = 0.19, which we broke down further to reveal more negative ERPs to *some*-infelicitous trials relative to *some*-felicitous trials across right fronto-central electrode sites, *t*(17) = 2.22; *p* = 0.04. Analyses on central-line electrode sites revealed a significant main effect of condition with brain activity being more negative to *some*-infelicitous than felicitous trials across central-line electrodes, *F*(1,17) = 4.93, *p* = 0.04, ηp2 = 0.23.

### Adults (*All*)

**FIGURE 5:**
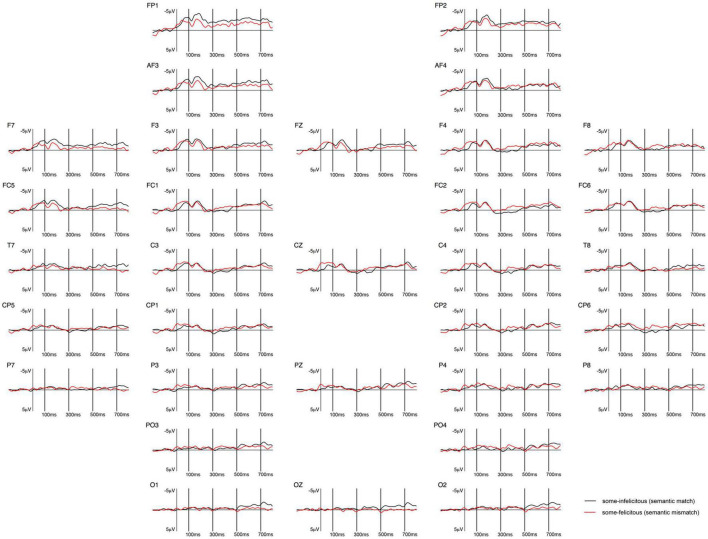
Grand-averaged ERPs recorded in adults, time-locked to the presentation of the word “Alle” (*all*) for the *some*-infelicitous condition (black line) compared to the *some*-felicitous condition (red line).

**FIGURE 6:**
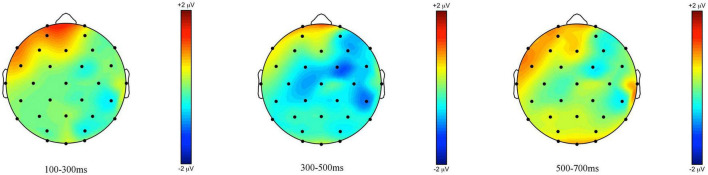
Topographic maps of the ERPs recorded in adults, time-locked to the presentation of the word “Alle” (all) for the some-felicitous condition minus the *some*-infelicitous condition in the three time windows.

The ERPs measured at the onset of the quantifier all in the *some*-felicitous vs. infelicitous conditions are plotted in Figure 5 and the topographic map are plotted in Figure 6. Visual inspection of the graphs suggests a left frontally distributed positivity between 300 and 500 ms in the *some*-felicitous condition relative to the *some*-infelicitous condition and a late positivity across occipital electrodes between 500 and 700 ms.

Analyses of the early time window revealed a significant interaction between condition and hemisphere across lateral electrode sites, *F*(1,24) = 5.53, *p* = 0.027, ηp2 = 0.19. Breaking down this interaction further revealed no significant modulation of brain activity across conditions in either hemisphere, ps > 0.1. No significant main effects or interactions with condition were found in the analyses on the central line electrodes in this time window, ps > 0.1.

In the 300–500 ms time window, analyses of the lateral electrodes highlighted a significant interaction between condition × hemisphere, *F*(1,24) = 4.5, *p* = 0.031, ηp2 = 0.18. Breaking the interactions with condition down, we found a significant difference in brain activity to *some*-felicitous and *some*-infelicitous trials across right centro-parietal electrodes, *t*(24) = -2.14, *p* = 0.042, with more negative brain activity to *some*-felicitous trials than *some*-infelicitous trials. No significant main effects or interactions with condition were found in the analyses on the central line electrodes in this time window, ps > 0.1.

In the 500–700 ms time window, analyses of the lateral electrodes highlighted a significant interaction between condition × hemisphere, *F*(1,24) = 5.64, *p* = 0.04, ηp2 = 0.16, and between condition × region × hemisphere, *F*(4,21) = 2.73, *p* = 0.056, ηp2 = 0.1. However, breaking these interactions down further revealed no significant modulation of brain activity by condition. Furthermore, no significant main effects or interactions with condition were found in the analyses on the central line electrodes in this time window, ps > 0.1. Nevertheless, based on visual inspection of the data, we also analysed brain activity across occipital electrodes in this time region. A 3 × 2 ANOVA with the factors laterality (left, central, and right) and condition (*some*-felicitous, *some*-infelicitous) yielded a significant main effect of condition, *F*(1, 24) = 4.32; *p* = 0.049; ηp2 = 0.15, with more positive brain activity to *some*-felicitous than *some*-infelicitous trials.

Furthermore, we analysed brain activity across frontal electrodes to assess the significance of the left-frontal positivity, which can be observed in Figure 6. A 2 × 2 ANOVA with the factors laterality (left: AF3, F3, F7, and Fp1; right: AF4, F4, F8, and FP2) and condition (*some*-felicitous and *some*-infelicitous) yielded significant interactions between condition × laterality in the three time windows (100–300 ms: *F*(1, 24) = 7.42; *p* = 0.012; ηp2 = 0.24; 300–400 ms: *F*(1, 24) = 6.67; *p* = 0.016; ηp2 = 0.22; 500–700 ms: *F*(1, 24) = 6.72; *p* = 0.016; ηp2 = 0.22). However, a main effect of condition was not revealed by any group or single electrode in any time window, which leads us to consider these effects with caution.

### Adults (*Some*)

**FIGURE 7:**
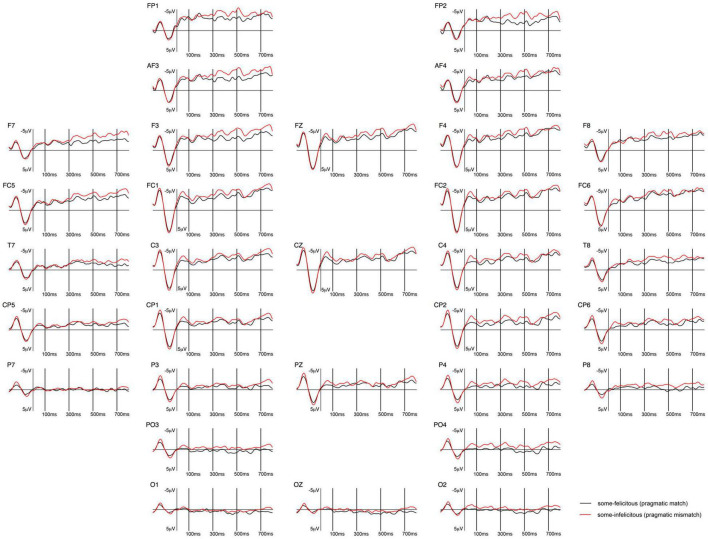
Grand-averaged ERPs recorded in adults, time-locked to the presentation of the word “Ein paar” (*some*) for the *some*-felicitous condition (black line) compared to the *some*-infelicitous condition (red line).

**FIGURE 8:**
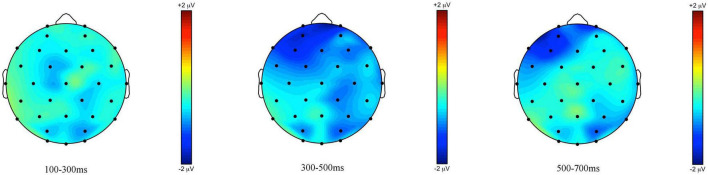
Topographic maps of the ERPs recorded in adults, time-locked to the presentation of the word “Ein paar” (*some*) for the *some*-infelicitous condition minus the *some*-felicitous condition in the three time windows.

The ERPs measured at the onset of the critical word *some* [*ein paar*], shown in Figures 7, 8, reveal a left frontally distributed negativity between 300 and 700 ms in the *some*-felicitous condition relative to the *some*-infelicitous condition.

Repeated measures ANOVAs on the early time window yielded no significant main effects or interactions with condition on either central line or lateral electrodes, ps > 0.1.

In the 300–500 ms time window, analyses of the lateral electrodes highlighted a significant interaction between condition × region × hemisphere, *F*(4,21) = 3.09, *p* = 0.045, ηp2 = 0.11. Pivoting on hemisphere, analyses on left hemisphere electrodes yielded a significant interaction between condition × region, *F*(4,21) = 4.79, *p* = 0.009, ηp2 = 0.17, and no significant main effects or interactions with condition across right hemisphere electrodes. Breaking this down further, we found a difference in brain activity to *some*-felicitous versus infelicitous trials, with more negative ERPs to *some*-infelicitous trials across left frontal, *t*(24) = 2.01, *p* = 0.055. No significant main effects or interactions with condition were found in the analyses on the central line electrodes in this time window, ps > 0.1.

In the 500–700 ms time window, analyses of the lateral electrodes highlighted a significant interaction between condition × region × hemisphere, *F*(4,21) = 4.06, *p* = 0.016, ηp2 = 0.15. Pivoting on hemisphere, analyses on left hemisphere electrodes yielded a significant interaction between condition × region, *F*(4,21) = 4.43 *p* = 0.012, ηp2 = 0.16, and no significant main effects or interactions with condition across right hemisphere electrodes. Breaking this down further, we found a significant difference in brain activity to *some*-felicitous versus infelicitous trials, with ERPs being more negative in *some*-infelicitous trials across left frontal electrode sites, *t*(24) = 2.22, *p* = 0.036. No significant main effects or interactions with condition were found in the analyses on the central line electrodes in this time window, ps > 0.1.

### Children and Adults

In order to further investigate the differences in ERP between children and adults we conducted an overall ANOVA including both groups. With *some* [*ein paar*], a significant interaction between condition × group was found in the central line electrodes in all the three time windows: 100–300 ms, F(1,41) = 5.39, *p* = 0.025, ηp2 = 0.12; 300–500 ms, F(1,41) = 7.89, *p* = 0.008, ηp2 = 0.16; 500–700 ms, *F*(1,41) = 5.69, *p* = 0.022, ηp2 = 0.12. The lateral electrodes yielded a four-way interaction between condition × group × region × hemisphere in every time window: 100–300 ms, *F*(1,41) = 10, *p* = 0.003, ηp2 = 0.20; 300–500 ms, *F*(1,41) = 6.28, *p* = 0.016, ηp2 = 0.13; 500–700 ms, *F*(1,41) = 6.71, *p* = 0.013, ηp2 = 0.14.

The ERPs measured at the onset of *all* [*alle*] yielded significant interactions in the central line between condition × group at 100–300 ms, *F*(1,41) = 4.80, *p* = 0.034, ηp2 = 0.10, and at 300–500 ms, *F*(1, 41) = 8.65, *p* = 0.005, ηp2 = 0.17. The lateral electrodes revealed a three-way interaction between condition × group × region at 100–300 ms, *F*(1,41) = 8.48, *p* = 0.006, ηp2 = 0.17, and between condition × group at 300–500 ms, *F*(1,41) = 4.23, *p* = 0.046, ηp2 = 0.09.

The more negative brain potentials to the scalar term some in *some*-infelicitous videos suggests that, 100 ms after the onset of the scalar term, participants were already sensitive to the felicity of the pairing of the auditory and visual stimuli (see Table 1 for a summary of the main results). The general pattern of more negative ERPs to *some* in a mismatching visual context (*some*-infelicitous videos) shows that both groups of participants, i.e., children and adults, were sensitive to the interpretation of these terms very early. In particular, children display the most robust effects on the central-line electrodes from the earliest time window whereas adults reveal a negativity with a more frontal-left topography. Furthermore, children display more positive ERPs to *all* in a mismatching visual context (*some*-felicitous videos) while adult controls gave rise to a weaker pattern of activations composed by a frontal-left positivity accompanied by an N400-like effect on right centro-parietal sites. This data support the conclusion that the effects elicited by the pragmatic violation are not due to general task-related strategies, such as merely associating a video with a word, or other underlying cognitive mechanisms, such as the violation of lexico-phonological expectations generated from the presentation of the visual context preceding the sentence.

**TABLE 1:** Summary of the statistical analysis from the ANOVAs conducted on children and adults.

	*Children–all [alle]*				
Time window	Electrodes	Effect	*F*	*p*-val.	Eff. size
100–300 ms	Central-line	Condition	4.67	0.045	0.22
100–300 ms	Lateral	Condition × region	2.39	0.048	0.15
300–500 ms	Central-line	Condition	6.74	0.019	0.28

	***Children–some [Ein paar]***				
**Time window**	**Electrodes**	**Effect**	***F*.**	***p*-val.**	**Eff. size**

100–300 ms	Central-line	Condition	5.43	0.034	0.24
100–300 ms	Lateral	Condition × region × hemisphere	2.951	0.067	0.15
300–500 ms	Central-line	Condition	8.2	0.011	0.32
300–500 ms	Lateral	Condition × region	2.61	0.043	0.13
500–700 ms	Central-line	Condition	4.93	0.04	0.23
500–700 ms	Lateral	Condition × region × hemisphere	3.13	0.02	0.16

	***Children and adults–some [Ein paar]***				
**Time window**	**Electrodes**	**Effect**	***F*.**	***p*-val.**	**Eff. size**

100–300 ms	Central-line	Condition × group	5.39	0.025	0.12
300–500 ms			7.89	0.008	0.16
500–700 ms			5.69	0.022	0.12
100–300 ms	Lateral	Condition × group × region × hemisphere	10	0.003	0.20
300–500 ms			6.28	0.016	0.13
500–700 ms			6.71	0.013	0.14

	***Children and adults–all [alle]***				
**Time window**	**Electrodes**	**Effect**	***F*.**	***p*-val.**	**Eff. size**

100–300 ms	Central-line	Condition × group	4.8	0.034	0.10
300–500 ms			8.65	0.005	0.17
100–300 ms	Lateral	Condition × group × region	8.48	0.006	0.17
300–500 ms		Condition × group	4.23	0.046	0.09

Instead, our results can be interpreted as indicating a difference in the reaction to different types of infelicity as previously found in other studies with adults that employed picture-sentence verification task and measured ERPs at the onset of the quantifier (cf. Politzer-Ahles et al., 2013; Panizza and Onea, 2014). This interpretation, however, must be considered with caution given that our study did not feature a full-fledged manipulation of semantic vs. pragmatic mismatches in the experimental design. Lastly, significant differences emerged between the two groups of participants (i.e., children vs. adult) and deserve a detailed explanation, which we will outline in the following paragraphs.

## Discussion

The current study set out to examine differences in children’s and adult’s access to the interpretation of scalar terms, i.e., *some* and *all*, and the speed with which such interpretations can influence processing. In particular, we examined participants’ brain responses to the scalar terms *all* and *some* across two situations. One, where participants heard the words *all* or *some* following a visual scenario where the critical character had some of a number of objects (*some*-felicitous contexts), and second, where participants heard the words *all* or *some* following a visual scenario where the critical character had all of a certain kind of object (*some*-infelicitous contexts).

Our results suggest that both children (aged 3-years) as well as adults show early and immediate sensitivity to the presentation of the scalar terms *all* and *some* in *some*-felicitous and infelicitous contexts. Brain activity to the critical word *some*, occurring in a statement following a visual presentation of the context and following a question about the quantity of objects including the quantifier *all*, was more negative in *some*-infelicitous relative to *some*-felicitous contexts, in both children and adult controls, despite the fact that the visual stimuli presented in both contexts were physically identical. In contrast, participants displayed a positivity to the term *all* occurring in a question when presented in *some*-felicitous relative to *some*-infelicitous context, which was early but sustained in children and late and posterior in adults.

There were, nevertheless, differences in the pattern of results found with adults and children. When looking at ERPs time-locked to *some* in the response, in children, the negativity was distributed in a similar way to the classic N400 component, i.e., maximum in central electrodes and attenuated in frontal and lateral sites. In contrast, in adults, the negativity was smaller in magnitude, at around 300 ms from the onset of *some* and more pronounced in the frontal electrodes of the left hemisphere, as attested by statistical analysis over the lateral electrodes and the overall ANOVA including both groups. Likewise, the positivity generated by *all* in the question was sustained across the three time windows, broadly distributed across central, centro-parietal and parietal electrode sites in children, while adults displayed an N400 with the same onset and distribution of the classic N400 component, namely maximum at centro-parietal sites of the right hemisphere (i.e., CP2 and CP6) and a late posterior positivity across occipital sites (i.e., O1, OZ, and O2). In what follows, we will discuss each of these results in more detail.

The early sensitivity to pragmatic felicitousness in ERPs to *some* displayed by children is consistent with previous reports of the timing and topography of the Phonological Mismatch Negativity (PMN; e.g., Connolly and Phillips, 1994) response to the phonological felicity of the pairing between auditory and visual stimuli. This component is typically thought to index acoustic-phonetic pre-processing of the sounds of an unexpected word in a given semantic context, in this case, sensitivity to the initial sounds of the word *some* in a context where use of *all* would have been more appropriate. We interpret this difference, therefore, as children’s sensitivity to the felicity of the word *some* in *some*-felicitous and *some*-infelicitous contexts; i.e., that children interpreted *some* as “some and not all”, and therefore infelicitous with a visual scenario where the participant was in possession of all of a certain kind of object. We note, however, this sensitivity continued into the N400 time window, with distinct topographical localisation of the effect across the two time windows, i.e., across central line electrodes in the early window and a left centro-parietal effect in the N400 time window. This is important since debate abounds as to whether these two components are functionally distinct (as claimed by Connolly and Phillips, 1994) and whether the PMN might, instead, be interpreted as an early onset N400 sensitive to word meaning comprehension (Mills et al., 2004; Sheehan et al., 2007; Lewendon et al., 2020), which can occur between 200 and 600 ms (Kutas and Federmeier, 2000).

As for the timing of this effect, significant as early as 100 ms after the presentation of the critical word *some* [*ein paar*] in children, we acknowledge that the present study does not let us rule out alternative explanations at the core of its nature. Our favoured interpretation maintains that this negativity is triggered by the online process of interpreting *some*--rapidly enriched via scalar strengthening to ‘‘some but not all’’--that generates a mismatch in the *some*-infelicitous contexts. However, given that our experiment did not employ filler sentences, the pragmatic violation could have been detected prior to the presentation of the critical word. Along with this hypothesis, the reported negativity could have been the result of a sustained negative trend that resisted baseline correction. Alternatively, it could be due to the re-activation of violation-related processes occurring earlier in time, which was elicited by hearing *some* [*ein paar*]^2^. It is important to remark that such alternative explanations are nonetheless consistent with the main finding of the present work, that is, the sensitivity of children to the pragmatic infelicity of *some* [*ein paar*].

In contrast, adults’ sensitivity to the scalar term in the prototypical N400 window (300–500 ms) had a frontocentral topographical distribution similar to that reported by previous auditory PMN/N400 studies (Connolly et al., 1990, 1992). Interpretation of this finding is, therefore, fairly simple with increased negativity to the scalar term in contexts where the alternative scalar term might have been more appropriate. The negativity, in this interpretation, merely indexes the ease of integration of the inappropriate scalar term in *some*-infelicitous contexts. In addition, given that this effect extends to the late time window (500–700 ms) it might be interpreted as a broadly-distributed negativity merging with a L-LAN. This component has been discussed in the literature in relation with semantic anomalies and re-analysis at the discourse/pragmatic level (cf. Panizza, 2012, for an overview).

Thus, like adults, children are able to access the scalar interpretation of the word *some*, i.e., *not all*, and display a sensitivity to the use of this word in *all*-contexts.

Taking the data from children and adults together, certain features of this sustained negativity, such as the frontal left distribution of the negativity elicited by adults and its early onset in children (although with distinct topographies across the two early time windows), may suggest caution in interpreting this negativity as a classic N400. On the other hand, we note that several other studies reported N400-like effects elicited by implicature mismatch with similar early onsets, e.g., Politzer-Ahles et al. (2013), Panizza and Onea (2014), and Nieuwland et al. (2010). For instance, Panizza and Onea report their N400 arising between 200 and 250 ms–earlier than 300–350 ms as usually found–and peaking far before the standard peak (i.e., 400 ms). Furthermore, we note that the central negativity detected in children manifests itself with a topography that is very similar to the N400 family, with its maximum at centro-parietal sites while it is attenuated at lateral and frontal ones. Indeed, the scalp topography of this effect does not seem to match that of the PMN, which is more frontally distributed (Connolly et al., 1990, 1992; Connolly and Phillips, 1994). Given these considerations, we are confident that the central negativity associated with *some* belongs to the family of N400-like effects that have been previously found in association with implicature violations (cf. Noveck and Posada, 2003; Nieuwland et al., 2010; Politzer-Ahles et al., 2013; Hunt et al., 2013; Panizza and Onea, 2014), in particular with regards to the negativity we report for children.

This being the case, we next discuss the contrast between the conclusions drawn above–of children’s sensitivity to scalar implicatures–and those of key previous studies to-date (Smith, 1980; Noveck, 2001; Papafragou and Musolino, 2003; Huang and Snedeker, 2009b; Barner et al., 2011; among many others). There are a number of reasons why our task might be more appropriate to tapping into children’s sensitivity to scalar implicatures. First, our task avoids presenting children with numerous visually similar referents–all children must do in the current study is display a sensitivity to the felicity of the match between a heard stimulus and a single visual referent. This might ease processing, in general, and allow children to display a keen sensitivity to the *some*-infelicitous trials. More importantly, our task provides a vital test of the hypothesis that children’s failure to retrieve the scalar interpretation in some tasks might be because of the absence of a suitable lexical alternative. Indeed, we show that, when provided with a suitable lexical alternative, children have no trouble retrieving the scalar interpretation of the word some and displaying sensitivity to its use in infelicitous contexts. In particular, when provided with the alternative scalar term all in the question *Has the hedgehog all the keys?* [*Hat der Igel alle Schlüssel?*], children are quick to detect the infelicitousness of the word some in contexts where the hedgehog does have all the keys. While this finding follows from the conclusions drawn from Barner et al. (2011), we show here that children are able to retrieve the scalar interpretation when provided with a suitable lexical alternative (as opposed to Barner et al., 2011).

Next, we discuss the positivity found in children and adults to *all* in *some*-felicitous relative to *some*-infelicitous contexts. The contrasting pattern of a sustained negativity to *some* and a sustained positivity to *all* (especially in children) across *some*-felicitous and infelicitous contexts provides further support for the computation of the implicature in both populations tested in the current study. As highlighted earlier, a very similar pattern of results was reported with the adults tested in Politzer-Ahles et al. (2013) using an analogous task (i.e., picture-sentence verification task) and measuring the ERPs at the onset of the quantifier (i.e., *some*/*all*). They found a broadly distributed positivity for semantic mismatch with *all*. Similarly, Panizza and Onea (2014) found a positivity associated with semantic violations elicited by all at 500 ms, which they interpreted as the reaction of the system to the semantic mismatch, i.e., semantic repair or detection of discourse complexity/anomaly (c.f. Kaan and Swaab, 2003 and Friederici et al., 2002). Taken together with the results from the current study, this consistent pattern of results suggests that the critical pattern of results obtained in the ERPs to *some* across felicitous and infelicitous contexts is not a result of a simple association between the words presented and the visual scenarios. We note, however, that we must be cautious in comparing our results with those from other studies, given the fact that, in our study, the strong quantifier was embedded in an interrogative clause, which does not constitute a typical semantic mismatch as in the other studies.

One interesting result emerging from the analysis exposed in the previous section is the N400 effect displayed at the onset of *all*, which was only found across right centro-parietal electrodes in adults in the 300–500 ms time window. We offer two explanations for this effect. First, it could be generated by the semantic mismatch between the context and the meaning of the quantifier, as found by Hunt et al. (2013) and Panizza and Onea (2014). Second, it could be due to the generation of a scalar implicature originated by the denial of *all* (i.e., “not all, thus some”). This second explanation suggests that adults consistently anticipated the implicature generation before hearing some in the some-infelicitous condition and would also provide an explanation for why the negativity they display with *some* is more attenuated in comparison to previous studies.

Summing up, we believe our study provides strong evidence supporting the claim that 3-year-old children are sensitive to scalar implicatures generated by *some*, and the violation thereof. This evidence is empirically linked to the sustained central negativity with early onset that we report in correspondence with *some* [*ein paar*] occurring after pragmatically infelicitous videos, and to the following related considerations:

• A similar negativity–but with different topography–was also found in adults, who are known to be sensitive to scalar implicature violation.
• It is unlikely to be the case that this effect is originated by the violation of auditory or lexical expectation of hearing some, given that *all*, argued to generate a similar expectation, elicited completely different ERP profiles (i.e., positivity instead of negativity in children vs. positivity/negativity in adults, associated with mismatch) in both groups.
• The negativity displayed by children does not have the typical topography of auditory-related effect such as PNM.
• The negativity has the same topography of other N400-like effects reported in previous studies investigating pragmatic violations and semantic anomalies.

These results extends what has already been found with older children (cf. Papafragou and Tantalou, 2004; Katsos and Bishop, 2011; Foppolo et al., 2012; Shetreet et al., 2014a) to 3-year-olds. One question that is relevant in this respect is whether this sensitivity is merely due to the knowledge that the weak quantifier *some* was misused in the infelicitous contexts or whether it requires a full-fledged computation of a quantity implicature. Our results, in line with what has been claimed in other works in developmental neuroscience (cf. Shetreet et al., 2014a) supports the hypothesis that they actually derived a scalar implicature that generated a mismatch at the level of the interpretation, affecting the N400 component. This conclusion brings important theoretical implications with respect to the acquisition of scalar implicatures, in that it allows us to rule out some hypotheses that account for why even older children often tolerate pragmatic violations in overt judgment tasks. Namely, children do not fail at computing scalar implicatures because of the lack of competence, cognitive resources or lexical knowledge of the relevant scales (Chierchia et al., 2001; Reinhart, 2004; Guasti et al., 2005; Barner et al., 2011; Skordos and Papafragou, 2012). Their tolerance to pragmatic violations is more likely due to the cognitive aspects related to overtly rejecting the infelicitous use of *some* such as cognitive task-related demands and conflict-monitoring difficulty as proposed by Papafragou and Musolino (2003), Papafragou and Tantalou (2004), Foppolo et al. (2012), and Shetreet et al. (2014a). We believe this conclusion can be empirically tested in further studies in two ways. One is to widen the sample of participants by including younger and older children and investigate the critical age at which children do not show this sensitivity through the same methodology we employed in our studies. The other way is to develop subtle and efficient decision tasks that are able to elicit pragmatic responses in 3-year-old children.

## Conclusion

In conclusion, we have demonstrated that at 3 years of life children are sensitive to violations generated by the infelicitous use of *some* [*ein paar*]. In addition, we found that children’s cognitive system seems to display such sensitivity rapidly, as rapidly as 100 ms from the auditory presentation of the word. One possible reason why our task was successful in this respect is that the scalar implicatures involved in our critical stimuli were extremely strong, as arising from a Question-Answer dialogue that put the scalar term on informational focus (i.e., at the focus of the QUD). We believe that this strategy can be fruitfully exploited by further studies to enhance children’s sensitivity to the phenomenon of scalar implicature derivation.

## Data Availability Statement

The datasets generated for this study can be downloaded at this link: https://osf.io/ng97b/.

## Ethics Statement

Ethical review and approval was not required for the study on human participants in accordance with the local legislation and institutional requirements. Written informed consent to participate in this study was provided by the participants’ legal guardian/next of kin.

## Author Contributions

DP wrote the manuscript and performed data analysis. EO helped revising the manuscript and designed the experiments. NM designed the experiments, and contributed writing the manuscript and analysing the data. All authors contributed to the article and approved the submitted version.

## Conflict of Interest

The authors declare that the research was conducted in the absence of any commercial or financial relationships that could be construed as a potential conflict of interest.

## Publisher’s Note

All claims expressed in this article are solely those of the authors and do not necessarily represent those of their affiliated organizations, or those of the publisher, the editors and the reviewers. Any product that may be evaluated in this article, or claim that may be made by its manufacturer, is not guaranteed or endorsed by the publisher.

## Footnotes

1 According to alternative accounts of scalar implicatures the meaning of such inferences is lexically encoded. Along the lines of the Grammatical View (cf. Chierchia et al., 2012) the scalar implicature is triggered by a silent operator whose meaning is that of the focus particle *only*, which can be optionally inserted in the sentence structure. Along with lexicalist views (c.f. Degen and Tanenhaus, 2015), instead, scalar triggers like *some* are ambiguous between a weak and a strong interpretation and optionality is accounted for by the optional choice of one of the two lexical entries.

2 The authors would like to thank a reviewer for highlighting this issue and providing us with insights.
