# Corrigendum: Early ERP Evidence for Children's and Adult's Sensitivity to Scalar Implicatures Triggered by Existential Quantifiers (*Some*)

**DOI:** 10.3389/fpsyg.2021.777595

**Published:** 2021-11-10

**Authors:** Daniele Panizza, Edgar Onea, Nivedita Mani

**Affiliations:** ^1^Department of English Studies, University of Göttingen, Göttingen, Germany; ^2^Courant Research Centre “Text Structures”, University of Göttingen, Göttingen, Germany; ^3^Department of German Studies, University of Graz, Graz, Austria; ^4^Psychology of Language Research Group, University of Göttingen, Göttingen, Germany; ^5^Leibniz ScienceCampus Primate Cognition, Göttingen, Germany

**Keywords:** pragmatics, implicatures in language acquisition, implicature, developmental pragmatics, pragmatic inferencing, speech processing, N400, scalar implicature

In the original article, there were mistakes in Figures 1–8, as published. The order of presentation of the figures was incorrect and did not correspond to the legend description. The corrected Figures 1–8, and corresponding captions, appear below.

**Figure 1:**
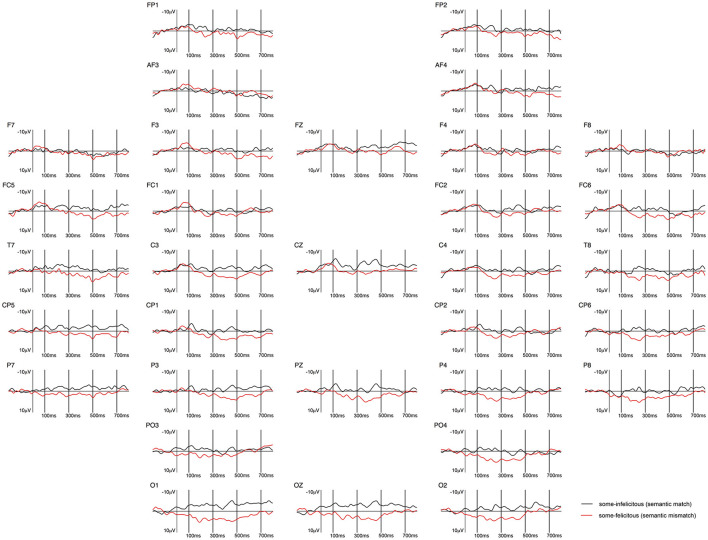
Grand-averaged ERPs recorded in children, time-locked to the presentation of the word “Alle” (*all*) for the *some*-infelicitous condition (black line) compared to the *some*-felicitous condition (red line).

**Figure 2:**
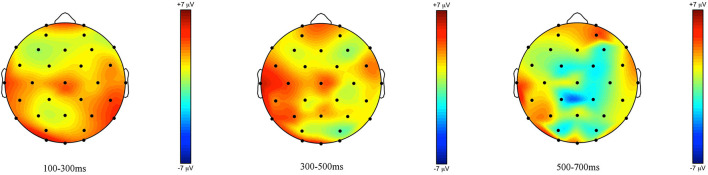
Topographic maps of the ERPs recorded in children, time-locked to the presentation of the word “Alle” (*all*) for the *some*-felicitous condition minus the *some*-infelicitous condition in the three time windows.

**Figure 3:**
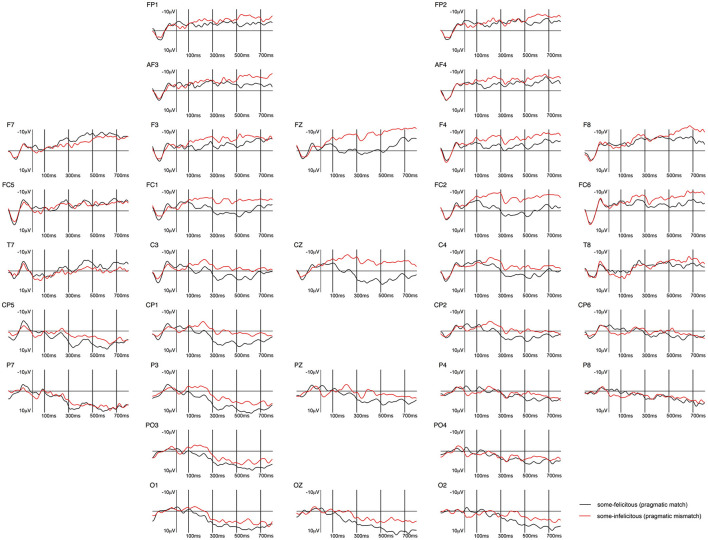
Grand-averaged ERPs recorded in children, time-locked to the presentation of the word “Ein paar” (*some*) for the *some*-felicitous condition (black line) compared to the *some*-infelicitous condition (red line).

**Figure 4:**
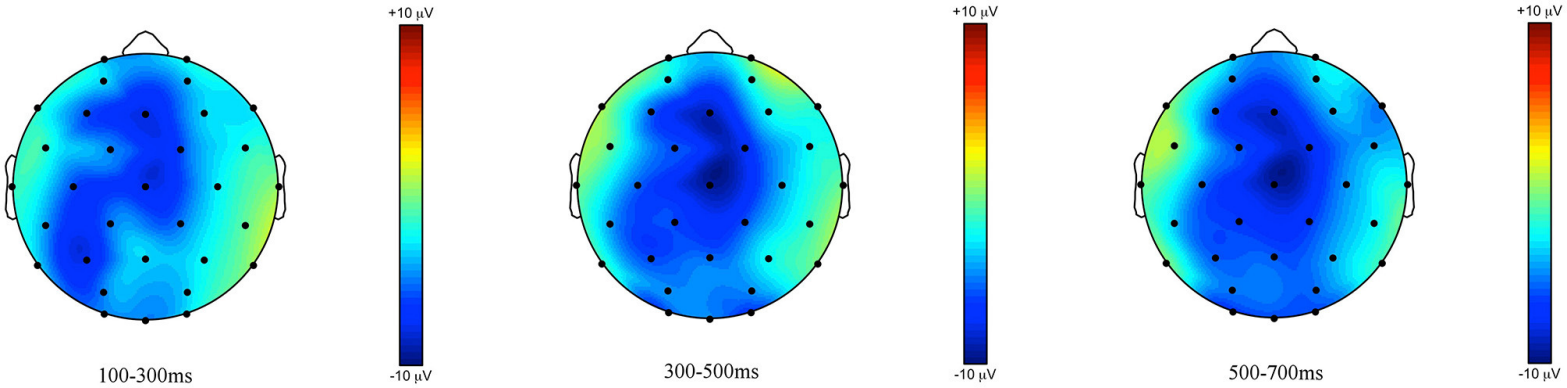
Topographic maps of the ERPs recorded in children, time-locked to the presentation of the word “Ein paar” (*some*) for the *some*-infelicitous condition minus the *some*-felicitous condition in the three time windows.

**Figure 5:**
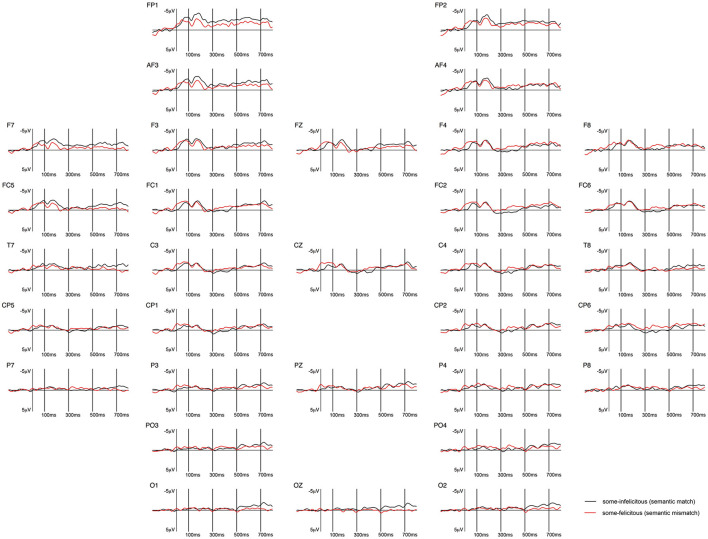
Grand-averaged ERPs recorded in adults, time-locked to the presentation of the word “Alle” (*all*) for the *some*-infelicitous condition (black line) compared to the *some*-felicitous condition (red line).

**Figure 6:**
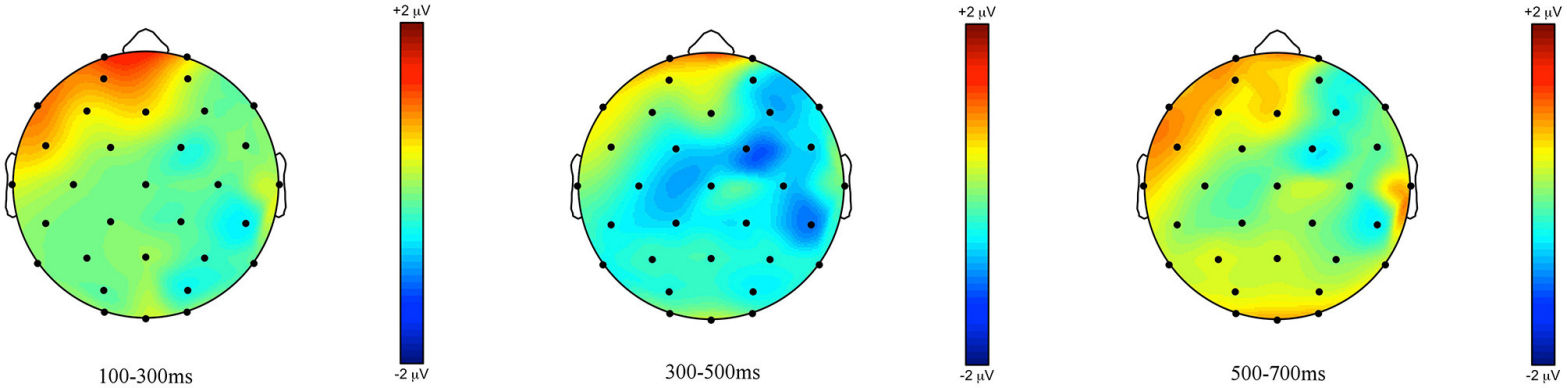
Topographic maps of the ERPs recorded in adults, time-locked to the presentation of the word “Alle” (*all*) for the *some*-felicitous condition minus the *some*-infelicitous condition in the three time windows.

**Figure 7:**
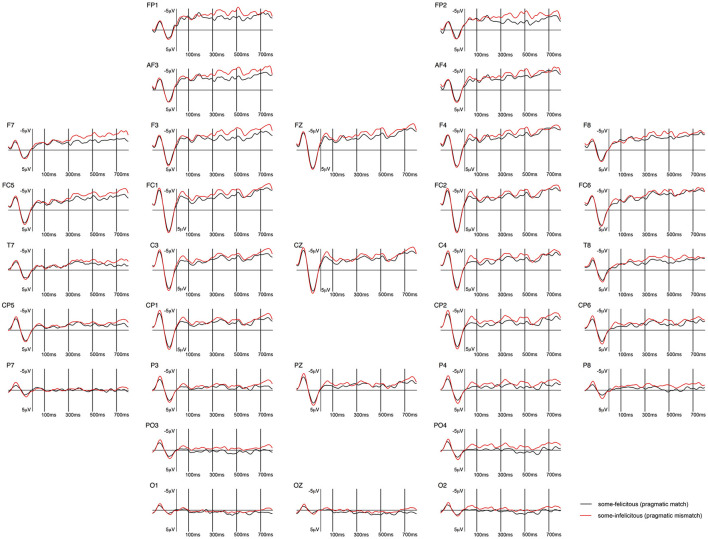
Grand-averaged ERPs recorded in adults, time-locked to the presentation of the word “Ein paar” (*some*) for the *some*-felicitous condition (black line) compared to the *some*-infelicitous condition (red line).

**Figure 8:**
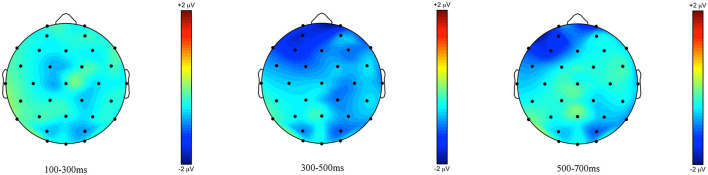
Topographic maps of the ERPs recorded in adults, time-locked to the presentation of the word “Ein paar” (*some*) for the *some*-infelicitous condition minus the *some*-felicitous condition in the three time windows.

The authors apologize for these errors and state that they do not change the scientific conclusions of the article in any way. The original article has been updated.

## Publisher's Note

All claims expressed in this article are solely those of the authors and do not necessarily represent those of their affiliated organizations, or those of the publisher, the editors and the reviewers. Any product that may be evaluated in this article, or claim that may be made by its manufacturer, is not guaranteed or endorsed by the publisher.

